# The Charitable Institutions of Victoria

**Published:** 1900-10-20

**Authors:** 


### THE CHARITABLE INSTITUTIONS OF VICTORIA.

According to the latest report issued by the Inspector of Charities, the number of subsidised charitable institutions and societies in Victoria in existence during 1899 was 77. This included 34 general hospitals, 6 special, and a convalescent home for men and another for women. The Royal Commission in 1891 had laid stress upon the unequal distribution of the Government grant, which had not then been allocated upon any defined principle. Of recent years great efforts have been made to gradually adjust the allocation, and an increased vote during the past year has enabled the treasurer to give each institution, as far as practicable, what it was entitled to compared with other institutions. Therefore it is now hoped that, allowing for special circumstances, the allocation is a very equitable one. The basis is the daily average of inmates; and when by chance, the average falls during the year, it cannot be avoided that for a few months at least the grant remains larger than the hospital actually requires. In addition to the ordinary maintenance grant the treasurer also allotted £10,000 for building purposes during 1899; but no institutions are for the future to have Government assistance towards any extensive building, without the sanction of the treasurer being first obtained towards the expenditure. This rule has been considered necessary because the committees of some of the institutions are found, by the visit of inspection, to lean towards the undertaking of superfluous building. A new ticket of admission, issued with the view of checking the amount of imposition in vogue at the hospitals, has been found to work very successfully. It prevents fraud, and does much towards insuring contributions from patients who, though not in a position to pay the ordinary medical fees, are able to afford a proportion. The new ticket was only in operation some two months before the close of the financial year, yet the improvement in the amount of patients' contributions was quite perceptible. The receipts for the twelvemonths 1897-8 had been £9,586; for the same period 1898-9 it was £11,508. Accordingly it is hoped that the new departure will, if properly carried out, materially increase the revenue of the hospitals and asylums. There is still room for improvement in the amounts of the municipal grants, and also in the contributions raised locally. The inspector especially remarks on the smallness of the amount raised by public entertainments—a source of revenue always responded to by the public, and which a large number of the institutions do not sufficiently utilise. The proportion of the cost of management to expenditure, of course, varies greatly. The highest is 49 per cent. at the Mildura, the lowest 26 per cent. at the Creswick Hospital, both being general hospitals. Amongst special institutions the Austin (incurables) heads the list with 34 per cent., the Echuca (consumptives) being at the bottom with 21 per cent. only. In addition to hospitals proper the Colony of Victoria has 9 institutions which are hospitals and benevolent asylums. In many of these the number of hospital cases largely exceeds the others, but no separate record is kept of the two classes, both being entered in the same book. This, it is hoped, will be altered in time.

